# Use of mycophenolate mofetil in patients with pediatric and adult primary nephrotic syndrome: information from a Japanese hospital claims database

**DOI:** 10.1007/s10157-022-02233-w

**Published:** 2022-05-17

**Authors:** Takashi Funatogawa, Yusuke Narita, Aya Tamura, Kazuma Mii, Yasuo Sugitani, Tomoaki Uchida

**Affiliations:** Chugai Pharmaceutical Co., Ltd., 1-1 Nihonbashi-Muromachi 2-Chome, Chuo-ku, Tokyo, 103-8324 Japan

**Keywords:** Hospital claims database, Nephrotic syndrome, Pediatric, Mycophenolate mofetil

## Abstract

**Background:**

Current treatment for frequently relapsing, steroid-dependent, or steroid-resistant nephrotic syndrome focuses on immunosuppressive therapies. Although the clinical guideline suggests the use of mycophenolate mofetil (MMF), limited information is available on patients with primary nephrotic syndrome who receive off-label treatment with MMF in Japan.

**Method:**

The dose, treatment duration, previous treatment, and characteristics of primary nephrotic syndrome patients receiving MMF were investigated using data from a Japanese hospital claims database (April 2008–September 2021).

**Results:**

Data on 424 primary nephrotic syndrome patients receiving MMF (146 patients < 18 years old; 278 patients ≥ 18 years old) were captured. The most common initial daily doses of MMF capsules (% of patients < 18 and ≥ 18 years old) were 1000 mg (31.9%, 36.8%), 1500 mg (16.0%, 23.8%), and 500 mg (23.6%, 17.3%), and the most common maximum daily doses were 1000 mg (43.8%, 32.9%), 1500 mg (23.6%, 28.9%), and 2000 mg (6.3%, 16.2%). Most patients (97.9%, 99.3%) were treated with a daily dose of 2000 mg or less. Among patients < 18 years old, the younger the patient, the lower the dose. MMF was used for more than 1 year in 30.8% of patients < 18 years old and in 28.8% of patients ≥ 18 years old.

**Conclusions:**

Our study suggested that off-label use of MMF for primary nephrotic syndrome has increased since 2012 in Japan. The dose of MMF used in patients with primary nephrotic syndrome was generally within the approved dose range for lupus nephritis and transplant-related diseases in Japan.

## Introduction

Idiopathic nephrotic syndrome is a disorder affecting the kidneys in which an excessive amount of protein passes through the glomerular filter, resulting in edema, hypoalbuminemia, and proteinuria, and which cannot be explained by currently known pathogenic drivers such as diabetes mellitus or vasculitis [1, 2]. The annual incidence of idiopathic nephrotic syndrome is 1.15–16.9 cases/100,000 children, varying by ethnicity and region [3]. In Japan, the estimated annual incidence of childhood-onset idiopathic nephrotic syndrome is 6.49 cases/100,000 children [4]. Adult-onset nephrotic syndrome is more etiologically heterogeneous compared to childhood-onset nephrotic syndrome [5]. In Japan, the number of pediatric and adult patients with primary nephrotic syndrome (including pediatric patients with idiopathic nephrotic syndrome) is about 16,000 [6].

Immunosuppressive therapies are important treatment for patients who have difficulty in treatment with steroid. The 2012 KDIGO Clinical Practice Guideline for Glomerulonephritis recommends cyclophosphamide (CYC), chlorambucil (CLB), levamisole (LEV), cyclosporine (CSA), or tacrolimus (TAC), and suggests mycophenolate mofetil (MMF) or rituximab (RTX) as corticosteroid-sparing agents for pediatric frequently relapsing nephrotic syndrome (FRNS)/ steroid-dependent nephrotic syndrome (SDNS) [7]. For adult patients, the guideline suggests CYC, calcineurin inhibitor (CSA or TAC), or MMF for frequently relapsing or steroid-dependent minimal change nephrotic syndrome (MCNS), CSA or MMF with high-dose dexamethasone for steroid-resistant focal segmental glomerulosclerosis (FSGS), and alkylating agent (CYC or CLB) or calcineurin inhibitor for idiopathic membranous nephropathy (IMN) resistant to initial therapy. Recently, the 2021 update to the KDIGO 2012 guideline has been published [8]. The guideline recommends alkylating agent, MMF, RTX, or calcineurin inhibitor as corticosteroid-sparing agents for pediatric patients with FRNS/SDNS, CYC, RTX, calcineurin inhibitor, or MMF for adult patients with frequently relapsing or steroid-dependent MCNS, calcineurin inhibitor for adult patients with steroid-resistant FSGS, and RTX, CYC, or calcineurin inhibitor for patients with IMN and risk factors for disease progression. In Japan, the 2020 pediatric clinical practice guideline of the Japanese Society for Pediatric Nephrology [9] recommends CYC or CSA and suggests RTX, mizoribine (MZR), MMF, or TAC for pediatric FRNS/SDNS. For adult patients, the 2020 adult clinical practice guideline of the Study Group on Intractable Kidney Diseases in Japan [10] suggests CSA, CYC, MZR, or RTX for frequently relapsing or steroid-dependent MCNS and FSGS, CSA or CYC for steroid-resistant MCNS, and immunosuppressive therapies including MMF for steroid-resistant FSGS and IMN resistant to initial therapy. In Japan, CYC and CSA are approved for both pediatric and adult FRNS or steroid-resistant nephrotic syndrome (SRNS), MZR is approved for adult SRNS, and RTX is approved for childhood-onset FRNS/SDNS. While the use of MMF is suggested in the global guideline [7, 8] and the Japanese guideline [9], MMF is not approved for either pediatric or adult patients with primary nephrotic syndrome.

Only limited information is available on primary nephrotic syndrome patients who received off-label treatment with MMF in Japan. Information from off-label use of MMF including the dose used in clinical practice is of particular importance since the dose of MMF is adjusted widely according to the patients’ conditions including their age and symptoms. In this study we extracted data from a Japanese hospital claims database and explored the dose, treatment duration, previous treatment, and characteristics of patients with a diagnosis of primary nephrotic syndrome who were treated with MMF.

## Material and methods

### Data source

To explore the dose, treatment duration, previous treatment, and characteristics of primary nephrotic syndrome patients treated with MMF in Japan, we used April 2008 to September 2021 data from the hospital claims database provided by Medical Data Vision Co. (MDV, Tokyo). The MDV database contains anonymized data on health insurance claims from diagnosis-procedure combination (DPC) hospitals in Japan; as of 2021, the database held data on about 36 million patients [11]. The conduct of this study was approved on 14 April 2021 by the research ethics committee, which is registered with the Ministry of Health, Labour and Welfare (Registration No. 11001059).

### Eligible population in the database

To identify patients with nephrotic syndrome, we extracted from the database those patients with the International Classification of Disease, 10th Revision (ICD-10) diagnosis code N04.x (nephrotic syndrome) [12]. To identify patients with primary nephrotic syndrome from among patients diagnosed with nephrotic syndrome, we excluded patients who could be identified as having secondary or congenital nephrotic syndrome at any time by using the following claim code data: congenital nephrotic syndrome (8836335), dense deposit disease nephrotic syndrome (8837974), secondary nephrotic syndrome (8838401), diffuse mesangial sclerosis (8849828), and Finnish congenital nephrotic syndrome (8849833). Furthermore, we excluded patients with the following ICD-10 diagnosis codes of typical secondary nephrotic syndrome at any time: N08.x (glomerular disorders in diseases classified elsewhere), which includes lupus nephritis and diabetic nephropathy, and N02.8 (IgA nephropathy). The date of primary nephrotic syndrome diagnosis was defined as the first day of the month in which the patient was first diagnosed with nephrotic syndrome. Lastly, patients who received the following transplantation were excluded using the Japanese procedure code for surgery; kidney transplantation (K780-0, K780-2), heart transplantation (K605-2, K605-4), liver transplantation (K697-5, K697-7), lung transplantation (K514-4, K514-6), pancreas transplantation (K709-3, K709-5, K709-6), and hematopoietic stem cell transplantations (K922-0, K992-2 [only from April 2008 to March 2010]). Patients classified as receiving MMF after the date of primary nephrotic syndrome diagnosis were defined as those patients who had received MMF after the diagnosis date and had not received MMF for at least 12 months before the diagnosis date.

## Results

### Patients

**Fig. 1:**
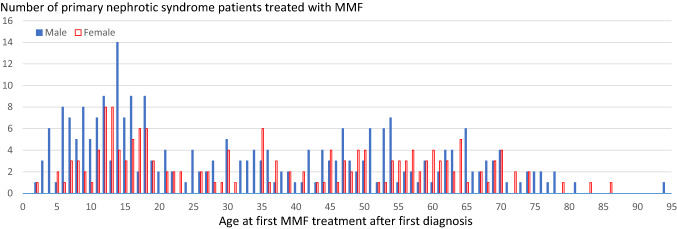
Number of primary nephrotic syndrome patients treated with MMF in the database *MMF*, mycophenolate mofetil

From the database, 424 patients were identified as having received MMF after the date of primary nephrotic syndrome diagnosis (146 patients < 18 years old, 278 patients ≥ 18 years old). Most patients did not have information about subtypes of nephrotic syndrome such as FRNS/SDNS (Appendix 1). The numbers of patients in these populations are shown in Fig. 1 by sex according to age. Among patients < 18 years old who were treated with MMF, there were more male patients (65.1%) than female patients (34.9%). Among patients ≥ 18 years old who were treated with MMF, the gender difference in the number of patients was smaller (males, 58.6%; females, 41.4%) than that of patients < 18 years old, and their ages at first MMF treatment after first diagnosis ranged widely.

Characteristics of primary nephrotic syndrome patients receiving MMF are shown in Table 1. For patients < 18 years old, the most common immunosuppressive therapies used after the diagnosis date up until the MMF initiation date were CSA (50.7%), MZR (26.7%), and RTX (21.9%). For patients ≥ 18 years old, the most common were CSA (15.1%), TAC (11.5%), and RTX (8.3%). Most patients (71.9% and 80.2% of patients < 18 and ≥ 18 years old, respectively) received oral steroid within 30 days prior to first MMF treatment. The mean daily oral steroid dose (mg prednisone equivalent) for 30 days prior to first MMF treatment was 18.7 and 7.8 mg in the patients < 18 and ≥ 18 years old, respectively. The numbers of patients who were < 18 years old and ≥ 18 years old at their MMF treatment after the date of primary nephrotic syndrome diagnosis are shown according to year (Table 2). Prescription of MMF for primary nephrotic syndrome increased after 2012, in which the global guideline [7] was issued, and has been stabilized in recent years.

**Table 1:** Characteristics of primary nephrotic syndrome patients receiving MMF

	< 18 years old^a^	≥ 18 years old^a^
*N*	146	278
Age, years, mean (SD)	11.10 (4.0)	47.42 (17.4)
Female, *n* (%)	51 (34.9%)	115 (41.4%)
Treatment prior to MMF^b^		
Cyclosporine	74 (50.7%)	42 (15.1%)
Mizoribine	39 (26.7%)	17 (6.1%)
Rituximab	32 (21.9%)	23 (8.3%)
Cyclophosphamide	4 (2.7%)	7 (2.5%)
Tacrolimus	3 (2.1%)	32 (11.5%)
Azathioprine	0	12 (4.3%)
None^c^	57 (39.0%)	185 (66.5%)
Daily oral steroid dose, mg, mean (SD)^d^	*N* = 105, 18.7 (15.3)	*N* = 223, 7.8 (7.0)

*MMF* mycophenolate mofetil, *SD* standard deviation

^a^Age at first MMF treatment after first diagnosis

^b^Treatment from first diagnosis until first MMF treatment. Some patients are counted several times as they received multiple immunosuppressive therapies

^c^“None” indicates patients who did not receive cyclosporine, mizoribine, rituximab, cyclophosphamide, tacrolimus, or azathioprine

^d^Daily oral steroid dose (mg prednisone equivalent) for 30 days prior to first MMF treatment. *N* indicates the number of patients who received oral steroid within 30 days prior to first MMF treatment, and mean and SD of daily oral steroid dose are derived from data of the patients who received oral steroid within 30 days prior to first MMF treatment

**Table 2:** Number of patients < 18 years old and ≥ 18 years old at the MMF initiation date after the date of primary nephrotic syndrome diagnosis, according to year

Year	Number of patients < 18 years old^a^	Number of patients ≥ 18 years old^a^
2008	0	0
2009	0	0
2010	1	6
2011	3	2
2012	3	7
2013	12	17
2014	23	31
2015	26	18
2016	13	26
2017	16	62
2018	17	34
2019	10	26
2020	12	25
2021^b^	10	24

*MMF* mycophenolate mofetil

^a^Age at first MMF treatment after first diagnosis

^b^From January to September 2021

### Dosage and treatment duration

Initial and maximum daily doses of MMF capsules in patients with primary nephrotic syndrome are shown in Table 3. In Japan, MMF oral suspension was additionally approved in 2015 for patients who cannot swallow the 250 mg capsules. The dose of MMF oral suspension is typically based on body surface area. In the database, we did not capture patients who received MMF oral suspension.

**Table 3:** Initial and maximum daily doses of MMF capsules for patients with primary nephrotic syndrome, according to age group

Age group^a,b^	< 18 years old										≥ 18 years old	
	All		0–4 years old		5–9 years old		10–14 years old		15–17 years old^c^		All	
*N*	144		10		40		62		32		277	
Dose (mg/day)	Initial	Maximum	Initial	Maximum	Initial	Maximum	Initial	Maximum	Initial	Maximum	Initial	Maximum
250	6 (4.2)	1 (0.7)	2 (20.0)	1 (10.0)	2 (5.0)	0	2 (3.2)	0	0	0	8 (2.9)	5 (1.8)
500	34 (23.6)	8 (5.6)	4 (40.0)	1 (10.0)	14 (35.0)	6 (15.0)	12 (19.4)	0	4 (12.5)	1 (3.1)	48 (17.3)	29 (10.5)
750	11 (7.6)	8 (5.6)	0	1 (10.0)	5 (12.5)	4 (10.0)	4 (6.5)	1 (1.6)	2 (6.3)	2 (6.3)	16 (5.8)	17 (6.1)
1000	46 (31.9)	63 (43.8)	1 (10.0)	2 (20.0)	11 (27.5)	18 (45.0)	23 (37.1)	34 (54.8)	11 (34.4)	9 (28.1)	102 (36.8)	91 (32.9)
1250	3 (2.1)	12 (8.3)	0	2 (20.0)	0	3 (7.5)	1 (1.6)	2 (3.2)	2 (6.3)	5 (15.6)	3 (1.1)	4 (1.4)
1500	23 (16.0)	34 (23.6)	0	0	0	6 (15.0)	14 (22.6)	19 (30.6)	9 (28.1)	9 (28.1)	66 (23.8)	80 (28.9)
1750	1 (0.7)	1 (0.7)	0	0	1 (2.5)	1 (2.5)	0	0	0	0	2 (0.7)	4 (1.4)
2000	6 (4.2)	9 (6.3)	0	0	0	0	4 (6.5)	5 (8.1)	2 (6.3)	4 (12.5)	30 (10.8)	45 (16.2)
2500	2 (1.4)	2 (1.4)	0	0	0	0	1 (1.6)	1 (1.6)	1 (3.1)	1 (3.1)	2 (0.7)	2 (0.7)
3000	1 (0.7)	1 (0.7)	0	0	0	0	0	0	1 (3.1)	1 (3.1)	0	0
Decapsulation required to administer the following doses^d^												
100	1 (0.7)	0	1 (10.0)	0	0	0	0	0	0	0	0	0
125	1 (0.7)	0	0	0	1 (2.5)	0	0	0	0	0	0	0
200	1 (0.7)	1 (0.7)	1 (10.0)	1 (10.0)	0	0	0	0	0	0	0	0
300	0	1 (0.7)	0	1 (10.0)	0	0	0	0	0	0	0	0
350	1 (0.7)	0	1 (10.0)	0	0	0	0	0	0	0	0	0
375	1 (0.7)	1 (0.7)	0	0	1 (2.5)	1 (2.5)	0	0	0	0	0	0
400	1 (0.7)	1 (0.7)	0	1 (10.0)	1 (2.5)	0	0	0	0	0	0	0
600	2 (1.4)	1 (0.7)	0	0	1 (2.5)	1 (2.5)	1 (1.6)	0	0	0	0	0
625	1 (0.7)	0	0	0	1 (2.5)	0	0	0	0	0	0	0
800	1 (0.7)	0	0	0	1 (2.5)	0	0	0	0	0	0	0
900	1 (0.7)	0	0	0	1 (2.5)	0	0	0	0	0	0	0

Values indicate the number of patients, and the values in parenthesis indicate the percentage

Prescription records with a daily dose of over 5000 mg were excluded owing to the high possibility of a data input error (total prescribed dose mistakenly entered as daily dose). In addition, records with two or more prescriptions on the same day were excluded owing to lack of accuracy. As a result, excluded from this table are two patients with only prescription records of over 5000 mg for daily doses (5700 mg for a patient aged 2 years and 11,000 mg for a patient aged 69 years) and one patient aged 11 years with only two prescription records on the same day.

*MMF* mycophenolate mofetil

^a^Age at first MMF treatment after first diagnosis

^b^Interpretation should be made cautiously, especially for young patients, owing to the possibility of decapsulation

^c^Japanese package insert recommends adult doses for patients ≥15 years old in general

^d^Patients whose prescription records showed a fractional amount of 250 mg capsules (e.g., 1.2 capsules) as the initial or maximum daily dose

The most common initial daily doses of MMF capsules (% of patients < 18 and ≥ 18 years old) were 1000 mg (31.9%, 36.8%), 1500 mg (16.0%, 23.8%), and 500 mg (23.6%, 17.3%), and the most common maximum daily doses were 1000 mg (43.8%, 32.9%), 1500 mg (23.6%, 28.9%), and 2000 mg (6.3%, 16.2%). Most patients (97.9%, 99.3%) were treated with a daily dose of 2000 mg or less. The maximum daily dose of 2000 mg was more frequent in patients ≥ 18 years old (16.2%) than in patients < 18 years old (6.3%). For patients < 18 years old, results in more detail according to age group are also described in Table 3; in general, the younger the patient, the lower the dose. There were only 10 patients aged from 0 to 4 years who were treated with MMF capsules. For patients aged from 5 to 9 years, the most common initial daily dose was 500 mg (35.0%) or 1000 mg (27.5%), and the most common maximum daily dose was 1000 mg (45.0%). For patients aged from 10 to 14 years and from 15 to 17 years, the most common initial daily dose was 1000 mg (37.1%, 34.4%), 1500 mg (22.6%, 28.1%) or 500 mg (19.4%, 12.5%), and the maximum dose was 1000 mg (54.8%, 28.1%) or 1500 mg (30.6%, 28.1%), respectively. In the database, we captured 11 patients whose initial daily doses and 5 patients whose maximum daily doses were recorded in fractional amounts of 250 mg capsules (for example, 1.2 capsules), and who were suspected to have carried out decapsulation.

Initial and maximum daily doses of MMF capsules in patients with primary nephrotic syndrome who received RTX prior to MMF treatment are shown in Table 4. In patients < 18 years old, the daily doses of MMF for patients who received RTX prior to MMF treatment was generally 1000 mg or less, and lower than those for all patients regardless of prior treatment shown in Table 3. In patients ≥ 18 years old, there were no clear difference in the same comparison.

**Table 4:** Initial and maximum daily doses of MMF capsules for patients with primary nephrotic syndrome who received RTX prior to MMF treatment, according to age group

Age group ^a,b^	< 18 years old										≥ 18 years old	
	All		0–4 years old		5–9 years old		10–14 years old		15–17 years old^c^		All	
*N*	31		3		6		16		6		23	
Dose (mg/day)	Initial	Maximum	Initial	Maximum	Initial	Maximum	Initial	Maximum	Initial	Maximum	Initial	Maximum
250	3 (9.7)	0	0	0	1 (16.7)	0	2 (12.5)	0	0	0	0	0
500	17 (54.8)	2 (6.5)	1 (33.3)	0	4 (66.7)	2 (33.3)	10 (62.5)	0	2 (33.3)	0	7 (30.4)	2 (8.7)
750	2 (6.5)	2 (6.5)	0	0	0	1 (16.7)	1 (6.3)	0	1 (16.7)	1 (16.7)	0	0
1000	5 (16.1)	22 (71.0)	0	1 (33.3)	0	3 (50.0)	3 (18.8)	15 (93.8)	2 (33.3)	3 (50.0)	8 (34.8)	9 (39.1)
1250	0	0	0	0	0	0	0	0	0	0	0	2 (8.7)
1500	1 (3.2)	2 (6.5)	0	0	0	0	0	1 (6.3)	1 (16.7)	1 (16.7)	3 (13.0)	4 (17.4)
1750	0	0	0	0	0	0	0	0	0	0	1 (4.3)	1 (4.3)
2000	0	1 (3.2)	0	0	0	0	0	0	0	1 (16.7)	4 (17.4)	5 (21.7)
Decapsulation required to administer the following doses^d^												
100	1 (3.2)	0	1 (33.3)	0	0	0	0	0	0	0	0	0
125	1 (3.2)	0	0	0	1 (16.7)	0	0	0	0	0	0	0
200	1 (3.2)	1 (3.2)	1 (33.3)	1 (33.3)	0	0	0	0	0	0	0	0
300	0	1 (3.2)	0	1 (33.3)	0	0	0	0	0	0	0	0

Values indicate the number of patients, and the values in parenthesis indicate the percentage

Prescription records with a daily dose of over 5000 mg were excluded owing to the high possibility of a data input error (total prescribed dose mistakenly entered as daily dose). In addition, records with two or more prescriptions on the same day were excluded owing to lack of accuracy. As a result, excluded from this table is one patient aged 11 years with only two prescription records on the same day

*MMF* mycophenolate mofetil

^a^Age at first MMF treatment after first diagnosis

^b^Interpretation should be made cautiously, especially for young patients, owing to the possibility of decapsulation

^c^Japanese package insert recommends adult doses for patients ≥15 years old in general

^d^Patients whose prescription records showed a fractional amount of 250 mg capsules (e.g., 1.2 capsules) as the initial or maximum daily dose

Duration of MMF treatment is shown in Table 5. Patients with > 1 year and > 2 years of treatment accounted for 30.8% and 12.3% of patients < 18 years old, respectively, and 28.8% and 14.7% of patients ≥ 18 years old, respectively. Among patients treated with RTX prior to MMF treatment, patients with > 1 year of MMF treatment accounted for 40.6% of patients < 18 years old and 8.7% of patients ≥ 18 years old.

**Table 5:** Duration of MMF treatment in patients with primary nephrotic syndrome, according to age group and prior treatment

Treatment duration (days)	< 18 years old^a^				≥ 18 years old^a^			
	Total (*N* = 146)	Treatment prior to MMF^b^			Total (*N* = 278)	Treatment prior to MMF^b^		
		Rituximab (*N* = 32)	Other ISTs^c^ (*N* = 88)	None^d^ (*N* = 57)		Rituximab (*N* = 23)	Other ISTs^c^ (*N* = 85)	None^d^ (*N* = 185)
< 10	15 (10.3)	1 (3.1)	7 (8.0)	8 (14.0)	34 (12.2)	6 (26.1)	10 (11.8)	22 (11.9)
10–29	14 (9.6)	3 (9.4)	6 (6.8)	8 (14.0)	39 (14.0)	5 (21.7)	16 (18.8)	20 (10.8)
30–59	11 (7.5)	1 (3.1)	4 (4.5)	7 (12.3)	33 (11.9)	1 (4.3)	11 (12.9)	22 (11.9)
60–89	7 (4.8)	3 (9.4)	4 (4.5)	3 (5.3)	21 (7.6)	2 (8.7)	5 (5.9)	14 (7.6)
90–119	5 (3.4)	2 (6.3)	1 (1.1)	3 (5.3)	19 (6.8)	2 (8.7)	9 (10.6)	10 (5.4)
120–179	14 (9.6)	4 (12.5)	10 (11.4)	4 (7.0)	23 (8.3)	1 (4.3)	7 (8.2)	16 (8.6)
180–364	35 (24.0)	5 (15.6)	23 (26.1)	12 (21.1)	29 (10.4)	4 (17.4)	13 (15.3)	16 (8.6)
365–729	27 (18.5)	9 (28.1)	19 (21.6)	8 (14.0)	39 (14.0)	1 (4.3)	8 (9.4)	30 (16.2)
730–1094	13 (8.9)	4 (12.5)	9 (20.2)	4 (7.0)	17 (6.1)	1 (4.3)	3 (3.5)	14 (7.6)
1095–1459	3 (2.1)	0	3 (3.4)	0	13 (4.7)	0	2 (2.4)	11 (5.9)
≥ 1460	2 (1.4)	0	2 (2.3)	0	11 (4.0)	0	1 (1.2)	10 (5.4)

Treatment duration was defined as days of continuous treatment with no more than six consecutive days of treatment withdrawal, regardless of the MMF formulation

Values indicate the number of patients, and the values in parenthesis indicate the percentage

*ISTs* immunosuppressive therapies, *MMF* mycophenolate mofetil

^a^Age at first MMF treatment after first diagnosis

^b^Treatment from first diagnosis until first MMF treatment

^c^“Other ISTs” includes cyclosporine, mizoribine, cyclophosphamide, tacrolimus, and azathioprine

^d^“None” indicates patients who did not receive rituximab or other ISTs

### Oral steroid dose

The daily oral steroid dose during the first 3-month MMF treatment period in patients with primary nephrotic syndrome is shown in Table 6. The results are based on the data of patients who did not receive any of the therapies shown in Table 1 (standard immunosuppressive therapies and RTX) during the first 3-month MMF treatment period. In patients < 18 years old, the daily oral steroid dose decreased gradually; the mean daily dose in the third month was about 35% of that in the first month. In patients ≥ 18 years old, the daily oral steroid dose was stable during the first 3-month MMF treatment period, irrespective of prior treatment of MMF. The daily oral steroid dose in the first month was lower in patients ≥ 18 years old than in patients < 18 years old.

**Table 6:** Daily oral steroid dose (mg prednisone equivalent) during the first 3-month MMF treatment period in patients with nephrotic syndrome

Post-MMF treatment		< 18 years old^a^				≥ 18 years old^a^			
		Total (*N* = 31)	Treatment prior to MMF^b^			Total (*N* = 18)	Treatment prior to MMF^b^		
			Rituximab (*N* = 6)	Other ISTs^c^ (*N* = 21)	None^d^ (*N* = 9)		Rituximab (*N* = 1)	Other ISTs^c^ (*N* = 11)	None^d^ (*N* = 7)
1st month	Mean	22.4	25.9	23.4	16.6	12.8	14.7	13.1	12.2
	Median	21.0	24.3	22.2	9.5	10.0	14.7	10.0	7.0
	SD	16.8	17.0	14.6	19.4	8.0	–	6.3	10.7
2nd month	Mean	12.3	4.2	11.4	15.7	10.4	9.9	10.9	9.5
	Median	8.3	1.8	9.3	5.8	9.4	9.9	9.9	7.0
	SD	15.0	5.4	11.3	22.2	6.2	–	5.2	8.0
3rd month	Mean	7.8	2.0	6.5	11.8	10.6	9.0	9.8	11.7
	Median	4.4	0.0	3.7	8.9	9.0	9.0	9.0	7.0
	SD	9.8	4.6	8.7	12.0	8.0	–	4.6	11.9

The results show the daily oral steroid dose (prednisone equivalent) of patients who received MMF treatment over 3 months and did not receive any of therapies shown in Table 1 (standard ISTs and RTX) during the first 3-month MMF treatment period. One month was calculated as 30 days

*ISTs* immunosuppressive therapies, *MMF* mycophenolate mofetil, *SD* standard deviation

^a^Age at first MMF treatment after the first diagnosis

^b^Treatment from first diagnosis until first MMF treatment

^c^“Other ISTs” includes cyclosporine, mizoribine, cyclophosphamide, tacrolimus, and azathioprine

^d^“None” indicates patients who did not receive rituximab or other ISTs

## Discussion

MMF was approved for graft-versus-host disease after hematopoietic stem cell transplantation in 2021 and lupus nephritis in 2016 in Japan in addition to its previous approval for organ transplant-related diseases. The approved dose is within the range from 500 to 3000 mg/day for adults (≥ 15 years old) and 300 to 1200 mg/m^2^/day for children (< 15 years old). The dose can be adjusted according to age and symptoms, but the maximum daily dose is 3000 mg for adults and 2000 mg for children. The dosage is based on the results of a survey conducted in Japan [13, 14] prior to the approval. The doses of MMF used in patients with primary nephrotic syndrome in the database were generally within the approved dose range for lupus nephritis and transplant-related diseases in Japan, and most patients were treated with a daily dose of 2000 mg or less.

A double-blind, randomized, placebo-controlled, multicenter trial (RCRNS01) conducted in Japan revealed that RTX treatment once weekly for 4 weeks is an effective and safe treatment, at least for 1 year, for childhood-onset complicated FRNS/SDNS [2]. Based on this result, RTX has become a standard therapy for pediatric patients with complicated FRNS/SDNS worldwide [15, 16]. However, since a certain proportion of patients who received RTX treatment tended to relapse after the recovery of B cell counts [2], a new maintenance therapy to prevent relapse after RTX treatment was needed. Recently, a double-blind, randomized, placebo-controlled trial (JSKDC07) conducted in Japan showed that remission-induction therapy with RTX followed by MMF as maintenance therapy can prevent treatment failure (defined as development of FRNS, SDNS, SRNS, or need for the use of other immunosuppressive agents or RTX) in most patients for a long period beyond the duration of peripheral blood B cell depletion by RTX [17]. The trial also showed that the effect of MMF was not sustained after discontinuation of MMF in the follow-up period. In our study, about 30% of patients received MMF treatment for > 1 year. In patients < 18 years old who were treated with RTX prior to MMF treatment, patients with > 1 year of treatment accounted for over 40%.

Other than RTX as prior treatment of MMF, positive results of MMF for CSA-dependent nephrotic syndrome [18] and relationship between mycophenolic acid blood levels and the efficacy after long-term CSA treatment for SDNS [19] have been reported in Japanese children. It has, however, been reported that MMF following CSA was not associated with improved long-term outcome of Japanese children with complicated SDNS [20]. In our study, over half of patients < 18 years old received CSA prior to MMF treatment.

This is the first study to investigate the actual usage of MMF for primary nephrotic syndrome in Japan using a large database which held prescription records on about 36 million patients, however, the present study had several limitations. First, we used DPC data in the MDV database, and data from local general practitioners of medicine were not included. The dosages used in DPC hospitals may differ from dosages used by general practitioners. The DPC data may include patients with more severe disease requiring treatment by more specialized medical doctors. Moreover, DPC data contain no data after patients transfer from the DPC hospital. This may bias the duration of MMF treatment toward shorter times. In the case that a patient transfers from one DPC hospital to another DPC hospital, the patient cannot be identified as the same patient. This may bias the number of patients toward a larger number. Second, claims data show only which drugs were dispensed, but not the deterministic prescribing information for a particular indication. While we excluded patients who were deemed to be treated with MMF within the approved indications, it is still possible that MMF was administered to treat other diseases in patients with primary nephrotic syndrome. Third, the claims-based definitions for primary nephrotic syndrome were not validated. Although we extracted patients with ICD-10 diagnosis code N04.x (nephrotic syndrome), similar to a previous study [12], and excluded patients who could be identified by ICD-10 diagnosis codes and claim code data as having secondary or congenital nephrotic syndrome, it cannot be ruled out that patients with secondary or congenital nephrotic syndrome were included. Furthermore, identification of subtypes of interest, such as FRNS/SDNS, was not possible because most patients did not have information about subtypes of nephrotic syndrome in the database as shown in Appendix 1. Fourth, it cannot be identified whether the purpose of MMF treatment is induction or maintenance from the database. The results of treatment duration should be interpreted cautiously. Fifth, the database does not include reliable data of body weight or body surface area. Hence, the results of doses per body weight or body surface area cannot be provided. Sixth, it cannot be ruled out that decapsulation might be carried out to adjust the doses for some young patients. Hence, the results about the doses in the 10 patients < 5 years old should be interpreted cautiously owing not only to the small number of patients but also to the possibility of decapsulation.

In conclusion, our study explored the off-label usage situation of MMF for pediatric and adult primary nephrotic syndrome in Japan. The dose of MMF used in practice varied depending on the condition of each patient but was generally within the approved dose range for lupus nephritis and transplant-related diseases in Japan.

## Appendix 1 Claim code data of primary nephrotic syndrome patients receiving mycophenolate mofetil

**Table:** 

ICD-10 diagnosis code^a^	Claim code data	Number of patients^b^
N04.0	8839471: nephrotic syndrome, minor glomerular abnormality	22
N04.2	8839551: nephrotic syndrome, diffuse membranous glomerulonephritis	2
N04.9	5819004: nephrotic syndrome	286
N04.9	8834799: pediatric nephrotic syndrome	20
N04.9	8835738: steroid-resistant nephrotic syndrome	87
N04.9	8838367: refractory nephrotic syndrome	53
N04.9	8839430: frequent relapse nephrotic syndrome	54
N04.9	8848070: steroid-dependent nephrotic syndrome	6
N04.9	8849711: primary nephrotic syndrome	1

^a^The International Classification of Disease, 10th Revision (ICD-10) diagnosis code

^b^Some patients are counted several times as they received multiple diagnoses at the same time
